# A population-specific reference panel empowers genetic studies of Anabaptist populations

**DOI:** 10.1038/s41598-017-05445-3

**Published:** 2017-07-20

**Authors:** Liping Hou, Rachel L. Kember, Jared C. Roach, Jeffrey R. O’Connell, David W. Craig, Maja Bucan, William K. Scott, Margaret Pericak-Vance, Jonathan L. Haines, Michael H. Crawford, Alan R. Shuldiner, Francis J. McMahon

**Affiliations:** 1 Human Genetics Branch, National Institute of Mental Health Intramural Research Program, Bethesda, MD 20892 USA; 2 Department of Genetics, Perelman School of Medicine, University of Pennsylvania, Philadelphia, PA 19104 USA; 3 Institute for Systems Biology, Seattle, WA 98109 USA; 4 Division of Endocrinology, Diabetes and Nutrition, Department of Medicine, University of Maryland School of Medicine, Baltimore, MD 21201 USA; 5 Neurogenomics Division, Translational Genomics Research Institute, Phoenix, AZ 85004 USA; 6 John P. Hussman Institute for Human Genomics, Miller School of Medicine, University of Miami, Miami, FL 33136 USA; 7 Institute for Computational Biology, Case Western Reserve University, Cleveland, OH 44106 USA; 8 Department of Anthropology, University of Kansas, Lawrence, KS 66045 USA

## Abstract

Genotype imputation is a powerful strategy for achieving the large sample sizes required for identification of variants underlying complex phenotypes, but imputation of rare variants remains problematic. Genetically isolated populations offer one solution, however population-specific reference panels are needed to assure optimal imputation accuracy and allele frequency estimation. Here we report the Anabaptist Genome Reference Panel (AGRP), the first whole-genome catalogue of variants and phased haplotypes in people of Amish and Mennonite ancestry. Based on high-depth whole-genome sequence (WGS) from 265 individuals, the AGRP contains >12 M high-confidence single nucleotide variants and short indels, of which ~12.5% are novel. These Anabaptist-specific variants were more deleterious than variants with comparable frequencies observed in the 1000 Genomes panel. About 43,000 variants showed enriched allele frequencies in AGRP, consistent with drift. When combined with the 1000 Genomes Project reference panel, the AGRP substantially improved imputation, especially for rarer variants. The AGRP is freely available to researchers through an imputation server.

## Introduction

Genetically isolated populations are subgroups that have remained reproductively separated from the surrounding population over many generations. Genetic isolates are often descended from a limited number of founding individuals and may suffer an increased burden of inherited disease. However, genetic isolates also offer many advantages for genetic studies^1^, so have long been of interest to geneticists^1–3^. Compared with outbred populations, population isolates generally have reduced genetic diversity due to founder effects^1, 4^. Individuals from isolated populations may also share more uniform environments, decreasing the non-genetic sources of variance contributing to complex traits. Some population isolates also have well-maintained genealogical records, which can facilitate family ascertainment and estimation of population parameters. Previous studies in population isolates have successfully identified genes and variants associated with Mendelian^5^ and complex traits^4, 6^.

Anabaptists represent a genetic isolate comprising several groups, including Amish and Mennonites, whose European ancestors settled in the Americas starting in the 17^th^ century^7^. Owing to religious conviction, cultural differences, and rural living, most Anabaptists have remained genetically distinct, despite rapid population expansion in recent decades^8, 9^. A number of otherwise rare Mendelian disorders have been identified among Anabaptists, with distinctive spectra of observed mutations characteristic of strong founder effects^10, 11^. High-throughput sequencing has renewed interest in the study of Anabaptist groups as a means of identifying genes underlying common, complex traits^9, 12^.

Since whole genome sequencing (WGS) is still expensive in large samples, many study designs rely on genotype imputation of unsequenced individuals. Several existing methods can use a framework of common variants (obtained, e.g., from a SNP array) to infer genotypes from local haplotypes represented within a reference panel of sequenced individuals^13^. Common alleles can be imputed with high accuracy, but imputation of rarer alleles can be challenging^13^. In isolated populations, imputation of rarer alleles can be improved by population specific reference panels that better represent founder alleles and haplotypes^6, 14^.

Here we present Phase 1 of the Anabaptist Genome Reference Panel (AGRP), the first whole-genome, population-specific imputation reference panel drawn from people of Anabaptist ancestry. The AGRP is built upon high-depth WGS data obtained from 265 individuals of Amish or Mennonite ancestry. We describe the population structure and allele frequency spectra represented by the AGRP and estimate the impact of this reference panel on the imputation accuracy of variants across the full range of allele frequencies.

## Results

### Sequencing and variant yield

Within each sequenced individual, we identified an average of 3.2 million autosomal variants, including 8,518 non-synonymous SNVs, 179 indels, and 183 putative loss of function (LoF) variants. In total, we identified ~12.3 million variants across all 265 Anabaptist individuals, including 993,567 indels and 2,458 putative LoF variants (Table 1). About 12.5% of the identified variants were novel (including ~11.1% of SNVs and ~21.9% of indels), defined here as variants not present in Kaviar^15^, dbSNP (v144) or ExAC (v0.3)^16^. Of all identified variants, 92,440 (0.75%) overlapped with protein-coding sequences; 47,270 (51.1%) of these were non-synonymous variants. Overall, ~18.5% of the identified variants were rare (MAF < 0.5%). However, a higher proportion of the non-synonymous variants (~28.1%), and an even higher proportion of the LoF variants (~50.9%) were also rare. This is consistent with negative selection.

**Table 1:** Discovered autosomal variants.

	Genome	SNV		Indels	LoF
		Synonymous	Non-synonymous		
**Total variants**					
Number of variants	12.3 million	40,416	47,270	993,567	2,458
Novelty rate^a^	12.5%	3.95%	7.96%	21.9%	35.5%
**Total variation by frequency** ^b^					
Common (MAF > 5%)	50.2%	45.8%	34.1%	41.9%	17.7%
Low frequency (MAF 0.5–5%)	31.2%	33.8%	37.8%	30.9%	31.5%
Rare (MAF < 0.5%)	18.5%	20.4%	28.1%	27.3%	50.9%
**Variants per individual**					
5^th^ percentile	3,178,457	9,242	8,097	189,720	166
Average	3,247,725	9,815	8,518	197,563	183
95^th^ percentile	3,325,349	10,059	8,734	203,980	200

^a^Versus Kaviar, dbSNP 144, and ExAC.

^b^Allele frequencies estimated in AGRP.

Genotype calls were highly consistent between duplicate samples and across genotype platforms. Genotype concordance rates calculated based on two pairs of duplicate samples were 99.9% for SNVs and 97.0% for indels and substitutions. SNV concordance rate was 99.7% within a subset of 46 individuals whose DNA was also genotyped with the Illumina OmniExpress Exome SNP array (no indels or substitutions are represented on this array).

Using the same SNP array data, we also estimated the sensitivity of the WGS platform to detect SNVs. Of the 640,931 high quality SNV sites identified by the array, 631,579 (98.5%) sites were also identified within our WGS data. The SNV detection sensitivity was similar across a range of MAF values (Figure S1).

**Figure 1:**
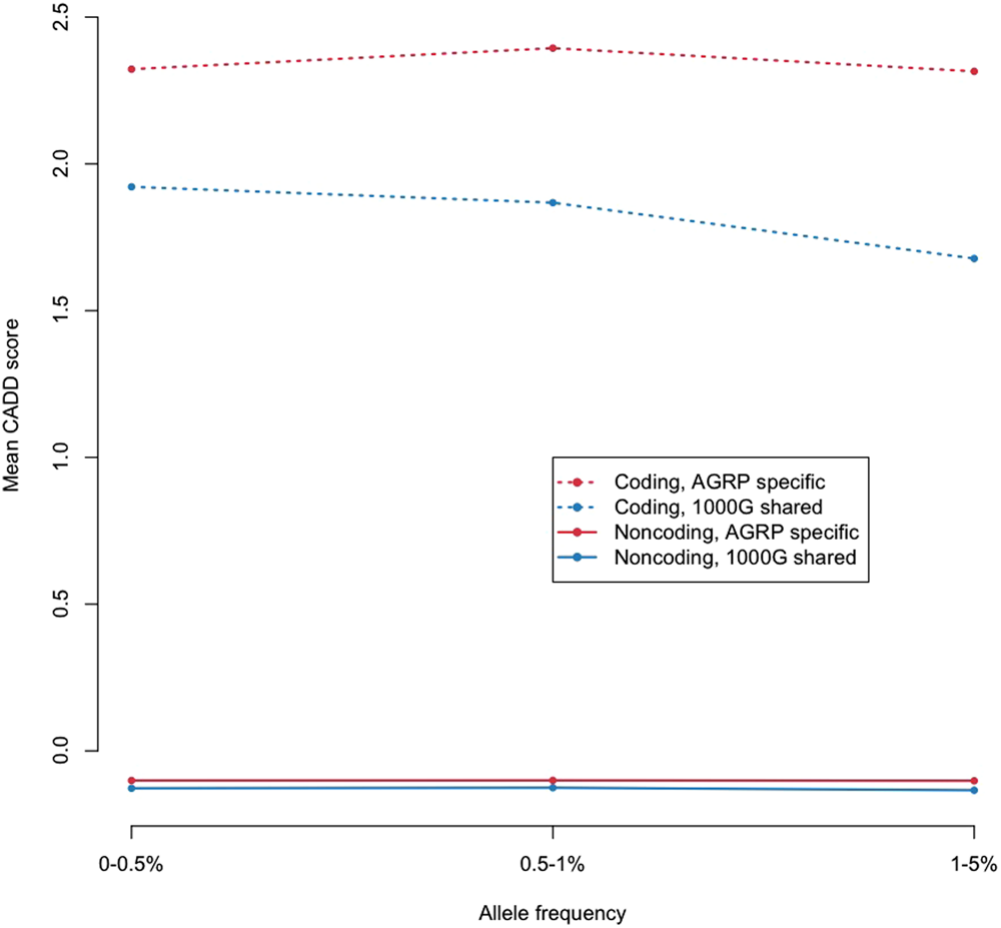
Variants seen only in AGRP are more deleterious. Mean Combined Annotation Dependent Depletion (CADD) scores for AGRP-specific variants (red) and those shared with the 1000G sample (blue), annotated as non-coding (solid lines) or coding (dotted lines), and binned by minor allele frequency.

Many Anabaptist populations suffer from high rates of otherwise rare Mendelian diseases^7^. Thus we investigated whether variants seen only in the AGRP were more deleterious, as estimated by the Combined Annotation Dependent Depletion (CADD) score^17^, than variants of comparable frequencies that were also identified in the 1000 Genomes Project sample. Indeed, both coding and non-coding variants seen only in the AGRP were significantly more deleterious (p < 0.0001; Fig. 1). The AGRP contains some individuals with psychiatric or metabolic disorders, so we repeated this analysis in only the healthy individuals. The results were similar (data not shown).

### Imputation

One of the main goals of this project was to provide a population-specific reference panel for genotype imputation in individuals of Anabaptist ancestry. Here we evaluated the accuracy with which variants of various MAF could be imputed. In order to illustrate the specific contribution of the AGRP to imputation accuracy, we reserved 10 individuals from the AGRP as a test subset and evaluated the results in four different reference panel configurations: (1) the AGRP alone; (2) the 1000G panel alone; (3) a merged panel comprising both 1 and 2; and (4) the Haplotype Reference Consortium (HRC) reference panel.

**Figure 2:**
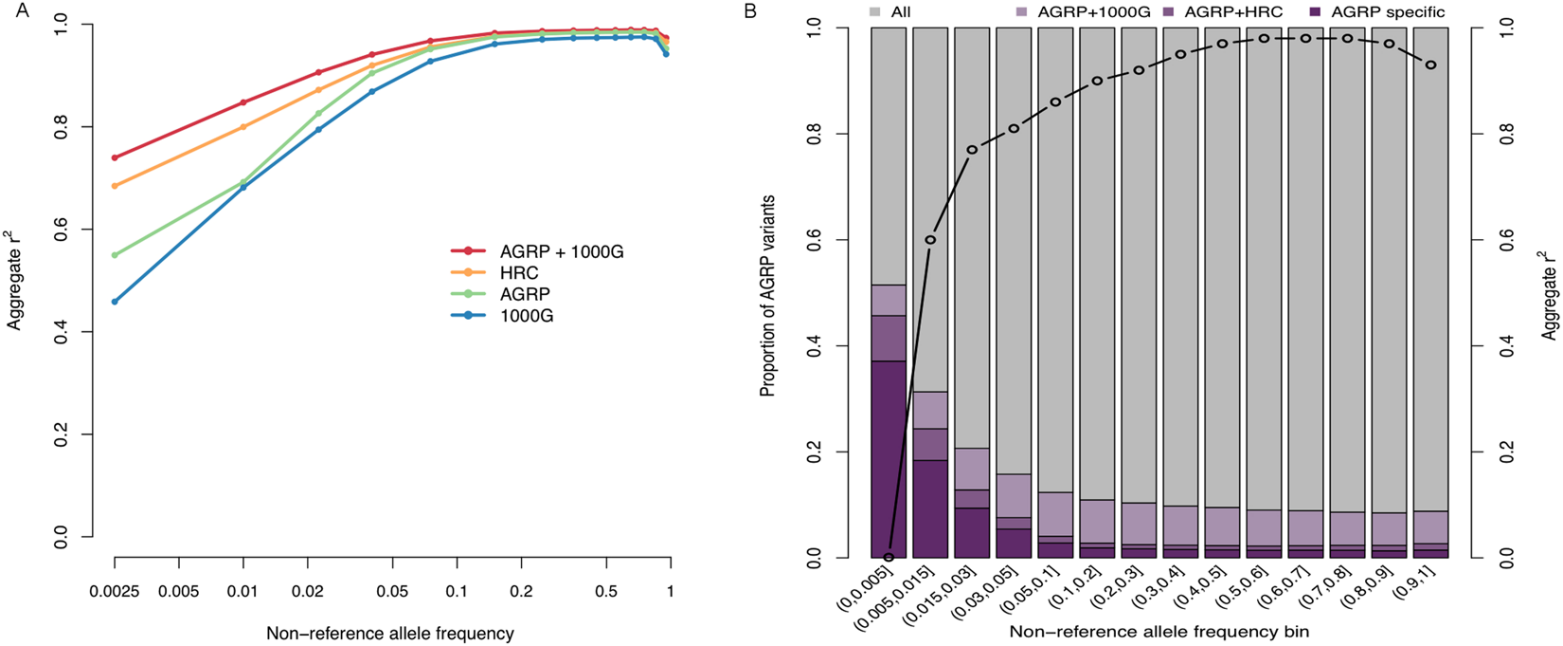
Imputation of shared and AGRP-specific variants. (**A**) Imputation accuracy (aggregate squared Pearson correlation coefficient, r^2^), across various reference panel configurations. The x-axis (log scale) shows the 1000G EUR (non-refernce) allele frequency of the variants being imputed. (**B**) The stacked bars show proportions of variants found in AGRP (left y-axis) that were also shared by various reference panels across a range of (non-reference) allele frequency bins in the AGRP. The embedded black line shows the imputation accuracy of AGRP-specific variants (right y-axis) when the merged AGRP/1000G reference panel was used.

The merged AGRP and 1000G reference panel provided the highest imputation accuracy across all allele frequency bins (Fig. 2A). The greatest advantage was observed for rarer alleles. For example, alleles with a 1000G MAF < 0.5% were imputed at an aggregate r^2^ of 0.74 with the merged AGRP/1000G panel, compared to 0.68 with the HRC panel, 0.55 with the AGRP alone, and 0.46 with the 1000G panel alone.

The AGRP also enabled imputation of a large number of variants apparently unique to Anabaptists. Overall, ~14.4% of variants identified in the AGRP were not present in the 1000G and ~18.3% were not present in the HRC panel. Although some (52.8%) of these Anabaptist-specific variants were very rare and thus un-imputable, the remaining variants could be imputed well, with aggregate r^2^ values in the 0.60–0.98 range (Fig. 2B).

### Genetic differentiation

**Figure 3:**
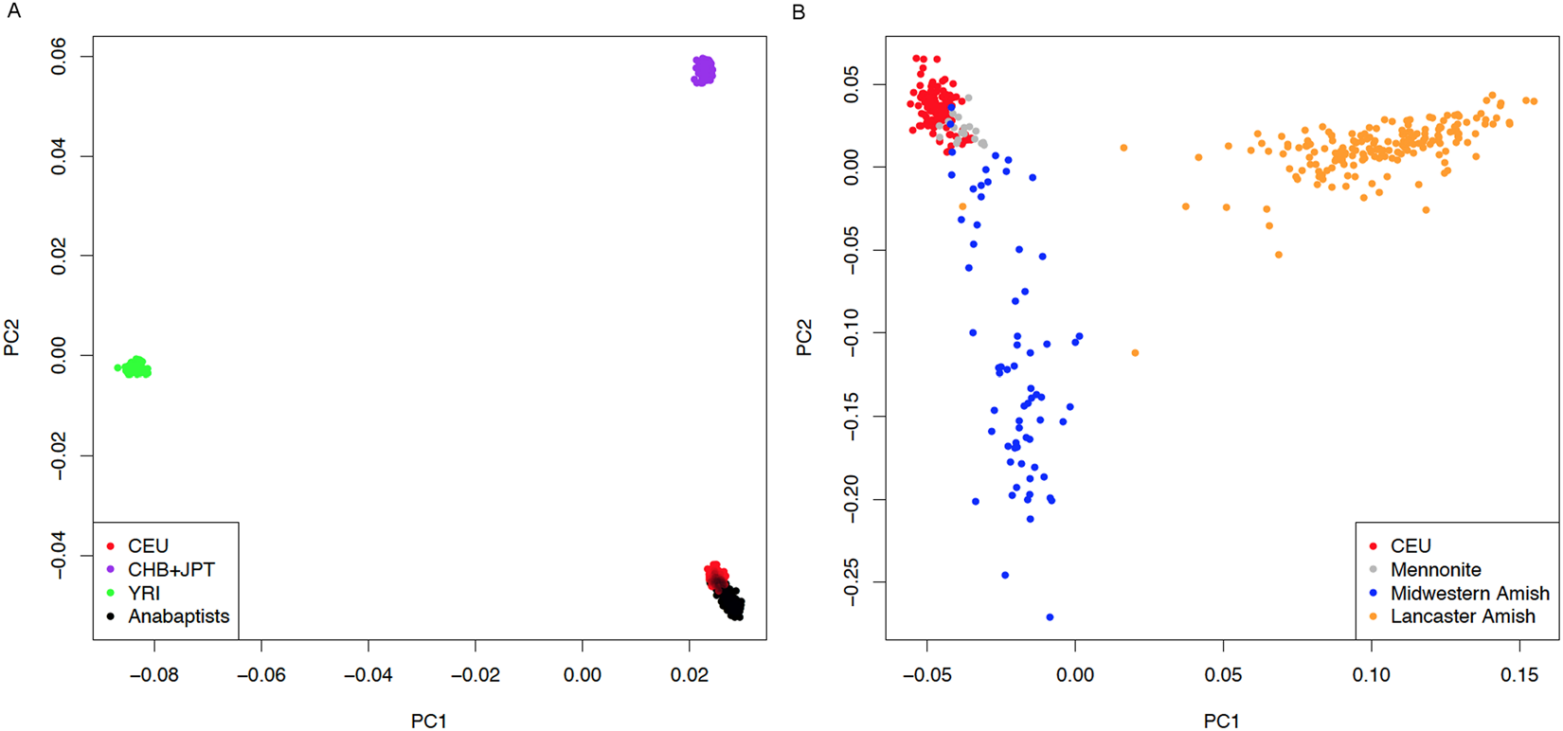
Plots of the first two principal components derived from the AGRP samples. (**A**) AGRP samples (black dots) are plotted along with European (CEU, red dots), Asian (CHB + JPT, violet dots), and African (YRI, green dots) samples taken from HapMap phase 3. (**B**) Mennonite (grey dots), Midwestern Amish (blue dots), and Lancaster County Amish (orange dots) within the AGRP, and CEU (red dots) from HapMap phase 3.

Principal components analysis (PCA) showed that individuals in the AGRP clustered with CEU participants from the HapMap project (Fig. 3), as expected. In agreement with the PCA results, the overall correlation of MAF values in the AGRP and the European-ancestry subset of 1000G was high (r^2^ = 0.97, Figure S2). For uncommon variants, however, the correlation was much lower. Variants with 1000G MAF < 5% were only weakly correlated with MAF estimated in AGRP (r^2^ = 0.24). Consistent with this result, 42,837 variants were rare or absent in 1000G (MAF < 0.5%) but reached frequencies >5% in AGRP (Table S1).

It is widely believed that founder effects (i.e., population bottlenecks and genetic drift) influence the structure of Anabaptist populations^8, 18^, but the magnitude of these influences is largely unknown. Since founder effects lead to depletion of variants that are rare in the ancestral population^19^, we sought to estimate the footprint of founder effects in today’s Anabaptists by assessing depletion of rare variants in AGRP. The numbers of variants detected in unrelated individuals in AGRP (defined as less than third-degree relatives by KING^20^) were compared to those detected in European-ancestry subsets of the 1000G panel that were matched to AGRP on sample size.

**Figure 4:**
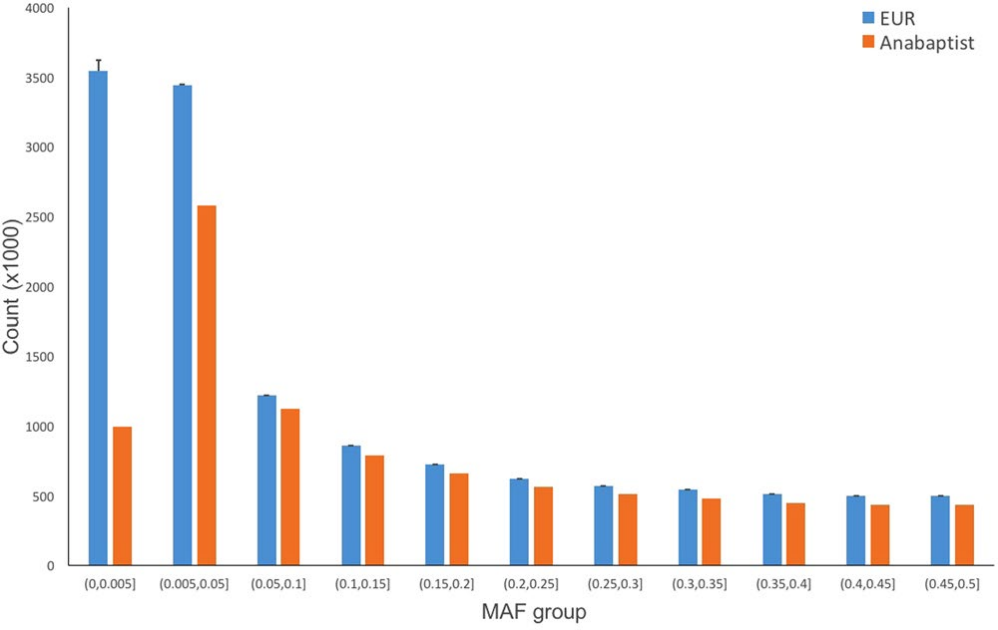
Variant counts by MAF group. The error bars for EUR (Europeans) were estimated based on 10 sub-samples of 102 randomly-selected Europeans in the 1000G sample.

A total of ~12.07 M rare variants (MAF ≤ 0.5%) are reported among the 500 unrelated European-ancestry samples in the 1000G panel. The subsets of 102 unrelated Europeans sampled from 1000G carried a mean of 3,545 K ± 77 K of these rare variants. Among the 102 unrelated Anabaptists in AGRP, however, only 995 K rare variants were identified (Fig. 4). This suggests a substantial depletion of rare variants in Anabaptists compared to outbred Europeans. The AGRP also contained significantly fewer variants with MAF between 0.5% and 5%, but the depletion compared to 1000G was modest. The number of common variants (MAF > 5%) was similar in both groups (Fig. 4).

**Figure 5:**
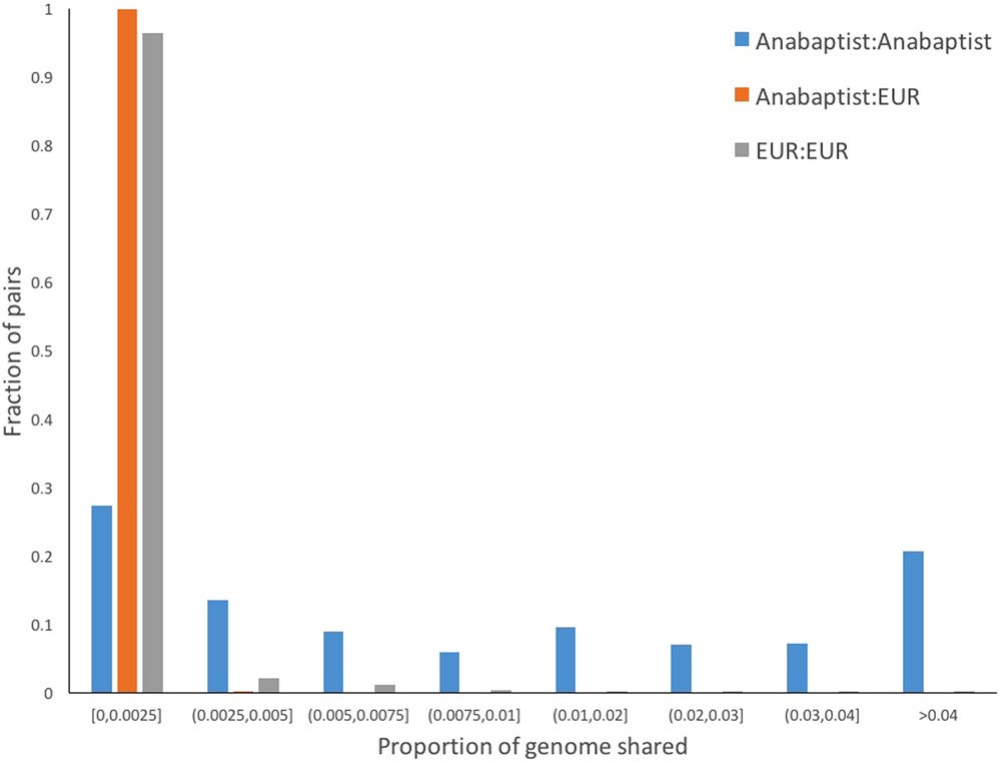
Increased distant relatedness among Anabaptists. The distribution, over all pairs of individuals, of the fraction of the genome shared IBD (segment lengths ≥3 cM) either within AGRP (Anabaptist, blue), within Europeans (EUR, grey), or between Anabaptists and EUR (orange).

The improved imputation accuracy conferred by the AGRP could be attributed, at least in part, to increased distant relatedness and reduced genetic diversity, both thought to characterize people of Anabaptist ancestry^8, 21^. To investigate distant relatedness, we compared the proportion of the genome shared identical by descent (IBD) among individuals within the AGRP to that shared between AGRP and ancestral Europeans in the 1000G. IBD sharing was substantially greater within the AGRP than within 1000G (Fig. 5): 44% of AGRP pairs shared at least 1% of the genome IBD, while >95% of 1000G pairs shared almost none of the genome IBD (<0.25%). Furthermore, very little IBD sharing (<0.5%) was detected between individuals in AGRP and individuals in1000G, demonstrating that the AGRP provides a sample of the ancestral European genome that is largely distinct from that represented by the1000G.

**Figure 6:**
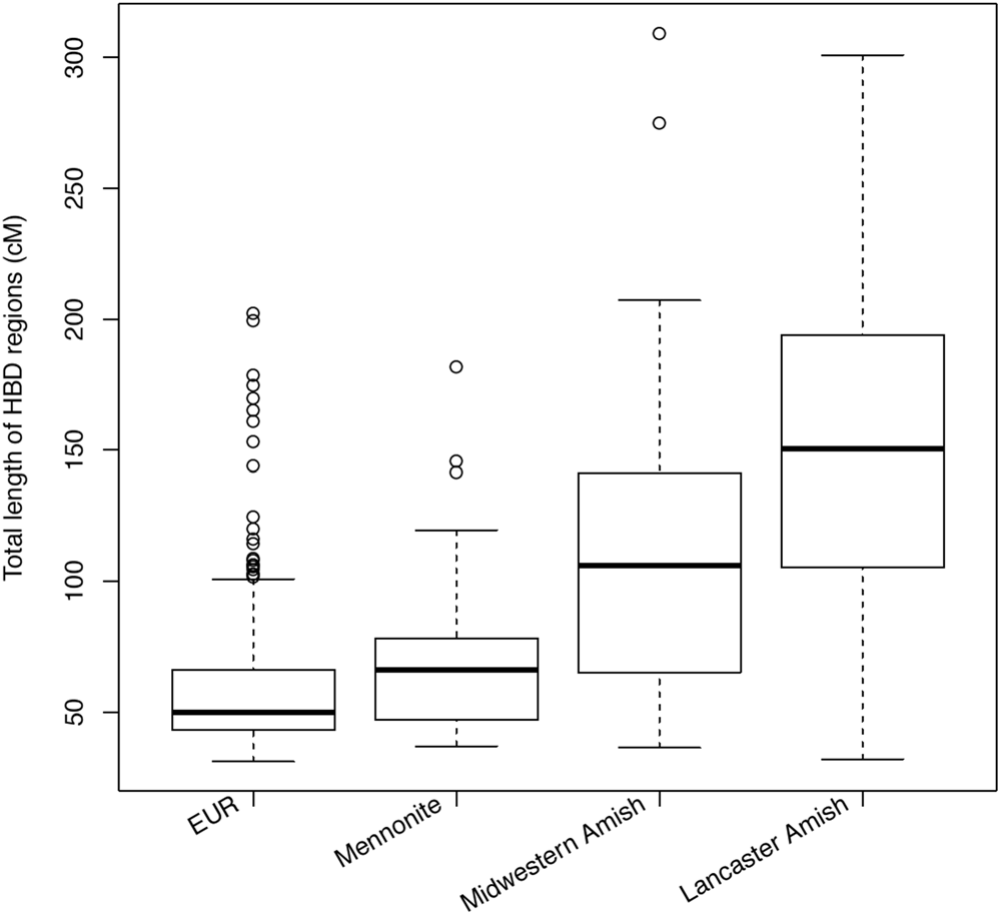
Reduced Genetic Diversity within Anabaptists. Total length of the genome homozygous by descent (HBD) within Europeans from the 1000G Project (EUR) and within various Anabaptist groups represented in the Phase I AGRP.

To investigate one measure of genetic diversity, we compared the total length of the genome shared homozygous by descent (HBD) among individuals within the AGRP to that shared between AGRP and Europeans in the 1000G. Total HBD length was significantly greater in AGRP than in 1000G (*P* < 0.001; Fig. 6). Total HBD length varied among the Anabaptist groups represented in the AGRP. The longest HBD regions were observed among Amish living in Lancaster County, Pennsylvania, followed by Amish living in the Midwestern United States. Mennonites in AGRP carried HBD regions that were only slightly longer on average than those observed in 1000G.

## Discussion

Imputation of SNP-array genotypes against a suitable reference panel represents an efficient and cost-effective strategy for investigating genetic variants not directly represented on inexpensive SNP arrays. However, current reference panels do not always perform well for rare variants and do not capture all of the rare variation present in human populations^22^. Our findings demonstrate the value of a population-specific reference panel for studies of Anabaptist founder populations. The AGRP effectively boosted imputation accuracy, especially for variants that are relatively rare. The increased imputability of rare variants seems to be explained by several key factors: 1) increased frequency of some otherwise rare alleles, presumably due to drift; 2) distant relatedness and decreased genetic diversity among Anabaptists, so that each rare allele is more likely to be represented on a single founder haplotype; 3) increased phasing accuracy due to family-relationship aware phasing methods and increased homozygosity by descent, which also increases the length of imputable haplotypes.

The AGRP performed best when merged with an existing large reference panel from the 1000G Project. Similar results have been observed in studies of other population-specific reference panels^23^. Increasing sample size will likely continue to improve imputation performance, consistent with what has been found by the 1000G Project^24^ and The Haplotype Reference Consortium^25^. Most important for studies of Anabaptist populations, the AGRP enables imputation of a large number of variants apparently unique to Anabaptists. We have shown that such variants tend to be more deleterious than variants of similar MAF that are also seen outside Anabaptist populations.

Footprints of the unique histories of Anabaptist populations are evident within the whole genome sequences represented by the AGRP. Common variants showed comparable allele frequencies in the AGRP and 1000G European panel, consistent with previous findings^26^. For rare variants, however, the allele frequencies were often quite different. Many variants rare or absent in the 1000G panel reached appreciable frequencies in the AGRP. On the other hand, many rare variants found in the 1000G panel were not detected in the AGRP, suggesting that these variants did not survive the population bottlenecks that occurred early in the Anabaptist population history or were lost due to random drift.

The substantial reduction of rare variants we observed in the AGRP is consistent with previous studies of other founder populations^19, 27^, but has not, to our knowledge, been previously demonstrated within Anabaptists. Since we did not sequence a comparable group of outbred Europeans in the present study, some of the differences we observed in the abundance of rare variants may be attributable to the sequencing platform and data analysis pipeline we used. However, it is unlikely that methodological differences alone can account for the substantial depletion of rare variants observed within the AGRP, since we detected no substantial difference in the sensitivity of variant calls across a broad range of minor allele frequencies (Figure S1).

Principal Components Analysis (PCA) analysis showed that the Anabaptist sample we studied was closely clustered with the Northern and Western European ancestry samples from HapMap. This is not surprising in light of the known recent European ancestry of Anabaptist groups. However, a finer scale PCA analysis was able to distinguish several different sub-populations within the AGRP, which corresponded roughly to known geographic and religious distinctions that have influenced Anabaptist mating patterns for most of the last 3 centuries^7^. Similar differences were also seen in the HBD analysis. These results suggest that the AGRP may perform better when used with studies of the Amish, but should still have value in other Anabaptist populations. The AGRP thus complements ongoing projects, such as TOPMed (G.R. Abecasis *et al*., 2015 ASHG, abstract) that include individuals drawn from the Amish communities in Lancaster, PA and surrounding areas.

This study has several limitations. First, the sample size is relatively small. This reduces representation of rare alleles and haplotypes. However, this only leads to underestimation of the value of the AGRP for imputation of rare alleles. The sample size also limits precision of allele frequency estimates, especially for alleles that are rare in Anabaptists. While important, this does not vitiate the AGRP for filtering variants that have drifted to high frequencies in Anabaptist populations and are thus unlikely to be highly deleterious. Second, allele frequencies should ideally be estimated separately for each subpopulation identified by the PCA analysis, but this will require a much larger sample size, especially for rare variants. Third, this Phase I of the AGRP mainly represents individuals of Amish ancestry. Mennonites, Brethren, Hutterites, and other Anabaptist groups are not well represented. Phase II of the AGRP is underway and will include a better representation of Mennonites, including those of Dutch-German ancestry (so-called “Russian Mennonites”.) Fourth, the AGRP is not a randomly selected sample of Anabaptists, but was based on sequences collected in the course of various common disease studies. While an epidemiological sample ascertained without regard to phenotype has advantages, inclusion of cases may actually increase the value of the AGRP for future studies focused on the same (or related) diseases, since any disease-associated alleles will presumably be better represented. Intuitively, inclusion of people with diseases caused by alleles of large effect could affect imputation of nearby alleles, but this is unlikely to be an important factor for the common, complex diseases represented within the AGRP. Indeed, a previous study concluded “No loss of imputation quality was observed using a panel built from phenotypic extremes”^28^.

We hope that the AGRP will facilitate a broad range of genomic studies in Anabaptist communities. The AGRP is available through an imputation server at https://www.nimh.nih.gov/labs-at-nimh/research-areas/clinics-and-labs/hgb/data-downloads.shtml.

## Methods

### Sample selection

WGS data were submitted by each of the collaborating groups, including: 106 individuals of Amish or Mennonite ancestry collected by the Human Genetics Branch, NIMH IRP and submitted by FJM, LH, and ISB (58 of whom suffered from a mood disorder), 81 individuals of Amish or Mennonite ancestry collected through the University of Maryland’s Amish Research Clinic in Lancaster, PA and submitted by ARS & JRO (38 of whom suffered from hypertension or a metabolic disorder); and 80 individuals of Amish ancestry collected by Janice Egeland and colleagues^12^ and submitted by MB & RLK (35 of whom suffered from bipolar disorder). All participants provided informed consent that explicitly allowed their coded genome sequencing data to be shared with other investigators. The final data set comprised WGS data from 267 individuals, including two pairs of duplicate samples that were used to evaluate sequencing and genotype accuracy.

### Sequencing and data generation

DNA derived from whole blood or from lymphoblastoid cell lines was obtained from the Rutgers University Cell and DNA Repository (Rutgers, NJ), the Corriell Institute (Camden, NJ), or the University of Maryland Amish Research Program. WGS was performed to >30× coverage by Complete Genomics, Inc. (CGI) using CGI’s combinatorial probe–anchor ligation (cPAL) method^29^. SNVs, insertions, and deletions were called with the CGI analysis pipeline version 2.0, 2.4 or 2.5 (described in refs 29, 30), relative to the human reference genome, GRCh37.

The CGA tools mkvcf command was used by each group to generate VCF files, which include all sites where at least one subject carried a non-reference allele. To confirm that the VCF files included the same types of variants and the genotype data had the same format, the same parameters (–source-names masterVar–field-names GT, FT, GQ, GL, DP, AD) were used by each group. The “–source-names masterVar” parameter asks the CGA tools to extract variants only from the masterVar files, so that only SNVs and short indels are included in the VCF files, while CNVs, SVs, and mobile element insertions (MEIs) are excluded. The “–field-names GT, FT, GQ, GL, DP, AD” parameter asks CGA tools to extract more information for each genotype call. Particularly, for non-reference genotype calls, CGA tools extracts GT (Genotype), FT (Genotype filters), GQ (Genotype Quality), GL (Genotype Likelihood), DP (Total Read Depth), and AD (Allelic depths) from the raw data. Previous studies have shown that false positive rates are higher for CNVs, SVs and MEIs than for SNVs in CGI data^14^, so these classes of variation were excluded from the reference panel.

### Quality control and variant annotation

All multi-allelic variants and variants located on non-autosomal chromosomes were removed from all three VCF files. All low-quality (marked by mkvcf as low quality; genotype quality <20; allele balance ratio <0.25 or >0.75 (applies to heterozygotes only); read depth <8) and half-called genotypes were set to ‘missing’. To make sure all variants were consistently represented in each VCF file, we normalized all VCF files using the VT variant tool set^31^. We then merged all VCF files and back-filled missing genotypes based on individual masterVar files. All Mendelian-inconsistent genotypes were set to missing. After QC, some variant sites no longer showed non-reference alleles; these were excluded. As the final QC step, variants with a missing rate >25%, Mendelian error rate >10% (based on 80 trios), or Hardy-Weinberg Equilibrium *P* < 1.0 × 10^−6^ were also excluded.

We assessed the accuracy of the sequencing data by: 1) comparing the genotype concordance rate within two pairs of duplicate samples; and 2) comparing the genotype concordance rate across 46 sequenced samples that were also genotyped with the Illumina OmniExpress Exome SNP array. We also used the same SNP array data to estimate the power of the sequencing pipeline to detect SNVs. Particularly, we calculated the fraction of high-quality variant calls (minor allele count >0, missing rate <5%, HWE *p* > 0.0001) detected by SNP array that were also identified in the CGI data.

All variants called in the AGRP were annotated with Combined Annotation Dependent Depletion (CADD) raw scores^17^, Ensembl Gene annotation, and 1000G (Aug 2015) allele frequencies, using ANNOVAR^32^. Variants were split into either ‘coding’ or ‘non-coding’ according to the Ensembl gene model, and categorized as “shared” if they were also identified in the 1000G. Otherwise variants were categorized as “AGRP-specific.” The Wilcoxon rank-sum test was used to test variants for association between sharing status and raw CADD scores. Since CADD scores are highly correlated with functional annotation and allele frequency^17^, separate comparisons were carried out for coding and non-coding variants within each of 3 AGRP allele frequency bins.

### Reference panel construction

To build the AGRP, we first phased the genomes with SHAPEIT2^33^. All known relationships were provided for phasing and the–duohmm option was enabled to make SHAPEIT2 incorporate pedigree information into the haplotype estimates, which has been shown to greatly improve the phasing accuracy^34^. In order to keep as many as haplotypes as possible but remove duplicate haplotypes for the final reference panel, we used KING^20^ to extract 131 individuals who were unrelated at the first-degree level. Thus 262 phased genomes were extracted to build the reference panel.

### Imputation

To assess imputation accuracy, we randomly selected a “test set” of 10 individuals from the AGRP. In the test set, autosomal genotypes were masked at all variable sites not represented on the Illumina Human OmniExpress SNP array. We chose this array since it represents an inexpensive and widely used array with over 700K SNPs common among people of European ancestry (http://www.illumina.com/products/human_omni_express_beadchip_kits.html).

Masked sites were then imputed by IMPUTE2 against: 1) the remaining AGRP, 2) the latest (phase 3) 1000 Genomes Project (1000G) reference panel alone, and 3) a merged panel comprising both the AGRP and the 1000G reference panel.

We also uploaded the phased VCF file of the test set to the Sanger Imputation Service (https://imputation.sanger.ac.uk/) and imputed the masked sites with the PBWT^35^ pipeline against the Haplotype Reference Consortium (r1.1) reference panel^36^. Variant sites were divided into 14 bins, based on MAF in the 1000G panel. Imputation accuracy was measured within each MAF bin as the aggregate squared Pearson correlation coefficient (r^2^) between imputed and actual allele dosages at the masked sites. To ensure comparability across the various reference panel configurations, only variants present in all panels were included in this comparison.

### Allele frequencies estimation and Population structure

The best linear unbiased estimator (BLUE)^37^, designed to estimate allele frequencies for complex pedigrees, was used to estimate the allele frequencies for all variants identified in the 265 Anabaptists. To infer population structure, principal-component analysis (PCA) was performed using ~100K LD-pruned SNPs. Since the sample included related individuals, we used a method called Principal Components Analysis in Related Samples (PC-AiR)^38^, which identifies principal components that accurately capture population structure, rather than family structure. We ran PC-AiR twice: once with HapMap Phase 3 data (from YRI, CEU, and CHB + JPT) and once with the CEU subset only. We did this to illustrate how AGRP individuals compare to the HapMap reference groups and to explore population structure within AGRP.

Founder effects may lead to depletion of rare variants^19^. To explore this phenomenon in the AGRP, we first extracted a list of unrelated individuals from the full set of 265 Anabaptists using KING^20^. We kept only one individual from each cluster of those with inferred relationships up to the 3rd degree, leaving 102 unrelated Anabaptists for this analysis. Using the same method, we also extracted all unrelated individuals from the European genomes in the final release of the 1000G Project, then randomly selected 102 of these for comparison. We compared the counts of variants in these 1000G individuals with counts in the 102 Anabaptists, across various allele-frequency bins. We performed this comparison 10 times by randomly selecting (with replacement) 102 individuals from the 500 unrelated Europeans in 1000G. By this procedure, we hoped to distinguish a true depletion of rare variants – which should be confined to variants with lower allele frequencies – from differences attributable to sequencing platform or variant calling pipeline, which should affect a broad range of allele frequencies, or differences due simply to sample size. We used only SNVs in this analysis, since other variant classes are called less accurately and are more sensitive to differences in sequencing platforms and pipelines.

### Identity-by-Descent (IBD) and Homozygosity-by-Descent (HBD)

Population isolates such as the Anabaptists often show increased IBD and HBD, owing to founder effects, inbreeding, and other population forces^2^. Such increases may increase imputation accuracy by improved phasing^39^. To assess these phenomena in the AGRP, we identified IBD and HBD regions with IBDseq^40^, a method designed specifically to identify such regions in WGS data. To compare the IBD sharing among Anabaptists, among other Europeans, and between Anabaptists and other Europeans, we merged WGS data from the AGRP with WGS data from the 503 other Europeans in the 1000G Project and used only the overlapping variants. We also compared the total length of HBD regions in both groups.

## Electronic supplementary material

Supplementary information

Table S1
