# Author Correction: A population-specific reference panel empowers genetic studies of Anabaptist populations

**DOI:** 10.1038/s41598-018-24604-8

**Published:** 2018-04-25

**Authors:** Liping Hou, Rachel L. Kember, Jared C. Roach, Jeffrey R. O’Connell, David W. Craig, Maja Bucan, William K. Scott, Margaret Pericak-Vance, Jonathan L. Haines, Michael H. Crawford, Alan R. Shuldiner, Francis J. McMahon

**Affiliations:** 1 Human Genetics Branch, National Institute of Mental Health Intramural Research Program, Bethesda, MD 20892 USA; 2 Department of Genetics, Perelman School of Medicine, University of Pennsylvania, Philadelphia, PA 19104 USA; 3 Institute for Systems Biology, Seattle, WA 98109 USA; 4 Division of Endocrinology, Diabetes and Nutrition, Department of Medicine, University of Maryland School of Medicine, Baltimore, MD 21201 USA; 5 Neurogenomics Division, Translational Genomics Research Institute, Phoenix, AZ 85004 USA; 6 John P. Hussman Institute for Human Genomics, Miller School of Medicine, University of Miami, Miami, FL 33136 USA; 7 Institute for Computational Biology, Case Western Reserve University, Cleveland, OH 44106 USA; 8 Department of Anthropology, University of Kansas, Lawrence, KS 66045 USA

Correction to: *Scientific Reports* 10.1038/s41598-017-05445-3, published online 20 July 2017

This Article contains errors in the Materials and Methods section under subheading ‘Sample selection’.

“All participants provided informed consent that explicitly allowed their coded genome sequencing data to be shared with other investigators”.

should read:

“Univ. of Pennsylvania samples were obtained from The Coriell Institute for Medical Research (CIMR). Collection of blood/tissue samples followed diagnostic consensus, using two informed consent forms: a) one with annual Univ. Miami IRB approval defining (with language appropriate for Old Order Amish) how their cells would be preserved for medical research on Major Affective Disorders; and, b) the Informed Consent Form required by the Institute for Medical Research (CIMR), later Coriell - National Institute for General Medical Sciences (NIGMS) Human Genetic Cell Repository (HGCR).The CIMR/NIGMS Informed Consent Forms makes no reference to specific diseases, types of genetic analysis that will be performed in future studies. Analysis of whole-genome re-sequencing data from consented individuals in this pedigree was also approved by the IRB of the Weill Cornell Medical College and the Perelman School of Medicine at the University of Pennsylvania. All other participants provided informed consent that explicitly allowed their coded genome sequencing data to be shared with other investigators”.

