# Supplementary material for: A population-specific reference panel empowers genetic studies of Anabaptist populations
Source: Sci Rep. 2017 Jul 20;7:6079. doi: 10.1038/s41598-017-05445-3 (PMC5519631; doi:10.1038/s41598-017-05445-3)
Supplement: Supplementary file 1 — Supplementary information [file 41598_2017_5445_MOESM1_ESM.pdf]

A population-specific reference panel empowers genetic studies of Anabaptist populations

Liping Hou,[1*] Rachel L. Kember,[2] Jared C. Roach,[3] Jeffrey R. O'Connell,[4] David W. Craig,[5] Maja Bucan,[2] William K. Scott,[6] Margaret Pericak-Vance,[6] Jonathan L. Haines,[7] Michael Crawford,[8] Alan R. Shuldiner,[4] and Francis J. McMahon[1*]

[1] Human Genetics Branch, National Institute of Mental Health Intramural Research Program, Bethesda, MD 20892 USA
[2] Department of Genetics, Perelman School of Medicine, University of Pennsylvania, Philadelphia, PA 19104 USA
[3] Institute for Systems Biology, Seattle, WA 98109 USA
[4] Division of Endocrinology, Diabetes and Nutrition, Department of Medicine, University of Maryland School of Medicine, Baltimore, MD 21201 USA
[5] Neurogenomics Division, Translational Genomics Research Institute, Phoenix, AZ 85004 USA
[6] John P. Hussman Institute for Human Genomics, Miller School of Medicine, University of Miami, Miami, FL 33136 USA
[7] Institute for Computational Biology, Case Western Reserve University, Cleveland, OH 44106 USA
[8] Department of Anthropology, University of Kansas, Lawrence, KS 66045-7556

*Correspondence: liping.hou@nih.gov

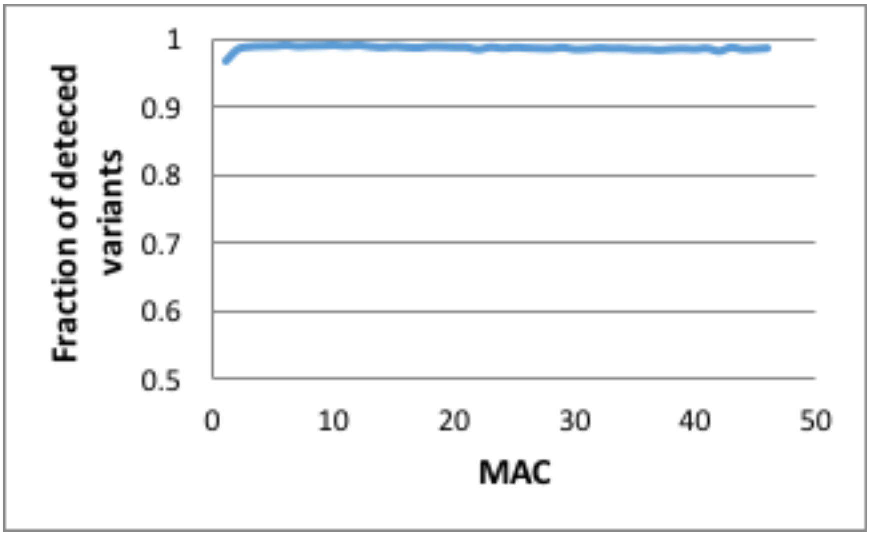

Figure S1. The sensitivity to detect SNVs measured as the fraction of detected variants. MAC: Minor Allele Count.

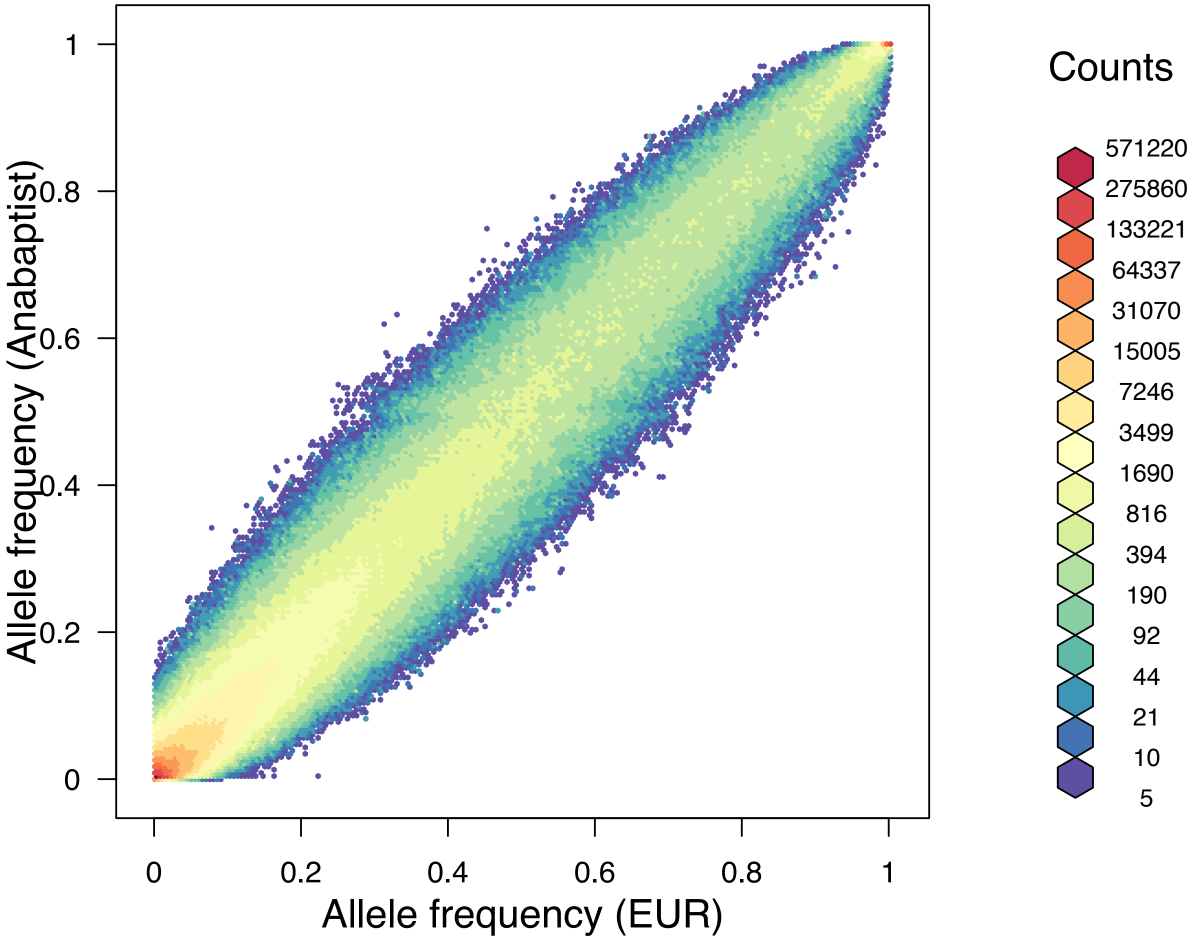

Figure S2. Density plot of the alternative allele frequencies for variants detected in the AGRP (Anabaptists, Y-axis) and European-ancestry individuals from the 1000 Genomes Project (EUR)
